# Butyrate enhances mitochondrial function during oxidative stress in cell lines from boys with autism

**DOI:** 10.1038/s41398-017-0089-z

**Published:** 2018-02-02

**Authors:** Shannon Rose, Sirish C. Bennuri, Jakeira E. Davis, Rebecca Wynne, John C. Slattery, Marie Tippett, Leanna Delhey, Stephan Melnyk, Stephen G. Kahler, Derrick F. MacFabe, Richard E. Frye

**Affiliations:** 1 Department of Pediatrics, University of Arkansas for Medical Sciences and Arkansas Children’s Research Institute, Little Rock, AR USA; 2 Kilee Patchell-Evans Autism Research Group, Alberta Children’s Hospital Research Institute, Calgary, AB Canada; 3 Division of Neurodevelopmental Disorders, Barrow Neurological Institute at Phoenix Children’s Hospital, Phoenix, AZ USA

## Abstract

Butyrate (BT) is a ubiquitous short-chain fatty acid (SCFA) principally derived from the enteric microbiome. BT positively modulates mitochondrial function, including enhancing oxidative phosphorylation and beta-oxidation and has been proposed as a neuroprotectant. BT and other SCFAs have also been associated with autism spectrum disorders (ASD), a condition associated with mitochondrial dysfunction. We have developed a lymphoblastoid cell line (LCL) model of ASD, with a subset of LCLs demonstrating mitochondrial dysfunction (AD-A) and another subset of LCLs demonstrating normal mitochondrial function (AD-N). Given the positive modulation of BT on mitochondrial function, we hypothesized that BT would have a preferential positive effect on AD-A LCLs. To this end, we measured mitochondrial function in ASD and age-matched control (CNT) LCLs, all derived from boys, following 24 and 48 h exposure to BT (0, 0.1, 0.5, and 1 mM) both with and without an in vitro increase in reactive oxygen species (ROS). We also examined the expression of key genes involved in cellular and mitochondrial response to stress. In CNT LCLs, respiratory parameters linked to adenosine triphosphate (ATP) production were attenuated by 1 mM BT. In contrast, BT significantly increased respiratory parameters linked to ATP production in AD-A LCLs but not in AD-N LCLs. In the context of ROS exposure, BT increased respiratory parameters linked to ATP production for all groups. BT was found to modulate individual LCL mitochondrial respiration to a common set-point, with this set-point slightly higher for the AD-A LCLs as compared to the other groups. The highest concentration of BT (1 mM) increased the expression of genes involved in mitochondrial fission (PINK1, DRP1, FIS1) and physiological stress (UCP2, mTOR, HIF1α, PGC1α) as well as genes thought to be linked to cognition and behavior (CREB1, CamKinase II). These data show that the enteric microbiome-derived SCFA BT modulates mitochondrial activity, with this modulation dependent on concentration, microenvironment redox state, and the underlying mitochondrial function of the cell. In general, these data suggest that BT can enhance mitochondrial function in the context of physiological stress and/or mitochondrial dysfunction, and may be an important metabolite that can help rescue energy metabolism during disease states. Thus, insight into this metabolic modulator may have wide applications for both health and disease since BT has been implicated in a wide variety of conditions including ASD. However, future clinical studies in humans are needed to help define the practical implications of these physiological findings.

## Introduction

The human body houses a diverse ecosystem of microbes collectively referred to as the human microbiome. Interestingly, it is believed that there may be more microbial cells than human cells in the human body with perhaps over 10–100 times more microbial genes than human genes^1–3^. The enteric (gut) microbiota is an area of great interest since it accounts for approximately 99% of the human microbiome^4^ and modulates the immune system^5^, metabolism^6^, receptor physiology^7^, and gene expression^8,9^. Alterations in the enteric microbiome, dynamically throughout the lifecycle, but particularly in early life, have been implicated in health and disease, including psychiatric disorders such as depression and anxiety^10^, gastrointestinal (GI) disorders^11^, inflammatory airway disease^12^, diabetes^13–15^, obesity^16,17^, atopic disease^5^, neurodegenerative conditions^18^, and early brain development and behavior^19–21^.

The microbiome modulates host physiology through the production of metabolic mediators, including lipopolysaccharides, peptidoglycans, short-chain fatty acids (SCFA), neurotransmitters, and gaseous molecules^22–24^. SCFAs, such as propionic acid (PPA), butyric acid (BT), and acetic acid, are produced as a consequence of fermenting carbohydrates and some proteins^18,25,26^ and modulate host physiology^26–28^. For example, PPA can modulate cell signaling^29,30^, cell–cell interactions^31^, gene expression^32,33^, immune function^34^, neurotransmitter synthesis and release^35^, and mitochondrial^36^ and lipid^37^ metabolism. PPA has positive health effects, having anti-obesity^27,38^, anti-inflammatory^27,38^, and anti-bacterial effects^39^, as well as lowering cholesterol^27^. Likewise, BT is a substrate for energy production, a regulator of energy metabolism^40^, a histone deacetylase inhibitor^41^, a modulator of immune function^42^, and a modulator of local gut physiology^43^. BT has positive effects in biological models of several important human diseases, including diabetes^43,44^, neurodegenerative disorders^18,45^, leukemia^46^, lymphoma^47^, and colorectal^48,49^, breast^50,51^, and pancreatic^52^ cancers.

Autism spectrum disorder (ASD) affects ~2% of children in the United States. The cause(s) of ASD are still unknown but evidence for a simple genetic defect is lacking^53^. The etiology of ASD likely involves environmental factors, which affect broad cell signaling, metabolic, immune, and epigenetic processes in genetically sensitive individuals^53,54^. Of particular note, ASD is associated with physiological disturbances including abnormal redox and mitochondrial metabolism. In fact, between 5% and 80% of children with ASD manifest mitochondrial dysfunction, with many demonstrating novel types of mitochondrial dysfunction rather than classic mitochondrial disease^37,55,56^. This is in comparison to the general population where mitochondrial disease is believed to effect <0.1% of the population^57^.

ASD is associated with GI and microbiome disturbances, potentially caused by an alteration in the dietary diversity, environmental exposures, C-section, antibiotics, formula feeding, and early hospitalization^2,20,36^. The enteric microbiome is uniquely positioned as a contributory etiological environmental factor for ASD^2^^,20,21,36^ and, as discussed in our recent review, there appears to be a close association between microbiome disturbances, mitochondrial dysfunction, and ASD^20^. We believe the microbiome is important to the understanding of ASD in some children since *Clostridia* spp, a major SCFA producer, has been repeatedly found to be overrepresented in the ASD gut^58,59^.

We have previously demonstrated that PPA may link the microbiome, mitochondria, and ASD. Brief pulsed PPA infusions into an adult rat cerebral ventricles produces behaviors and physiological changes associated with ASD^25^^,26,60^, while rats systemically exposed to PPA pre- and post-natally develop ASD-like behaviors in a sexually dimorphic manner, recapitulating the neurodevelopmental aspects of ASD^61–63^. Additionally, the metabolic changes found in ASD patients have been replicated in the PPA rodent model. Of note, a unique pattern of biomarkers of mitochondrial dysfunction found in the PPA rodent model is found in 17–24% of the children with ASD^37,64^. Furthermore, PPA modulates mitochondrial respiration in a concentration- and exposure time-dependent manner in lymphoblastoid cell lines (LCLs) derived from children with ASD^65^.

Another major SCFA produced by the gut microbiome, BT, also has connections to ASD and mitochondrial function. BT rescues behavior^66,67^ and brain pathophysiology^67^ and pathology^66,68^ in animal models of ASD. BT supports mitochondrial function, stimulating oxidative phosphorylation and fatty acid oxidation^69^, upregulates physiological stress pathways^69–71^, and is critical for colonocyte mitochondrial function in germ-free mice^40^. Animal models have suggested that BT, given through the GI tract or injected intraperitoneally, may be therapeutic in neurologic and psychiatric conditions including depression^72^, dementia^73–75^, traumatic brain injury^76^, and motoneuron disease^77^. However, early post-natal exposure to BT may contribute to colitis^78^.

Given the connection between ASD and mitochondrial function and given that BT is a fermentation product of ASD-associated bacteria^26^, we believe that BT is an important SCFA to study. We applied our LCL model of mitochondrial dysfunction in ASD to help understand the role of BT in modulating mitochondrial function. We have developed a cell line model of ASD in which LCLs from children with autistic disorder (AD) are classified into two groups: those with normal mitochondrial function (AD-N) and those with atypical mitochondrial function (AD-A)^79–81^. The AD-A LCLs have respiratory rates approximately twice that of the control (CNT) and AD-N LCLs and are very sensitive to in vitro increases in reactive oxygen species (ROS)^79–81^. Given the positive modulatory effects of BT on mitochondrial function, we hypothesized that BT would improve mitochondrial function in AD-A LCLs while having little effect on the CNT and AD-N LCLs. Furthermore, given the ability of BT to increase the expression of cellular pathways involved in cellular stress, we anticipated that this effect would extend to improving mitochondrial function in the AD-N and CNT LCLs when they are subjected to increased physiological stress.

Thus, we measured the effects of BT on mitochondrial function and determined whether these effects are dependent on concentration, exposure duration, the microenvironment redox state, or the underlying mitochondrial physiology (i.e., LCL group: AD-A, AD-N, CNT). In addition to studying how these factors modulated mitochondrial function on average in the overall groups, we examined the effect of BT on modulating mitochondrial function on an individual cell line basis. Furthermore, we measured the ability of BT to modulate the expression of key genes linked to mitochondrial and physiological stress as well as genes linked to cognition and behavior in ASD.

## Methods

### LCLs and culture conditions

ASD LCLs were derived from white boys diagnosed with AD chosen from pedigrees with at least other 1 affected male sibling (i.e., multiplex family) [mean (SD) age 8.5 (3.0) years]. These LCLs were obtained from the Autism Genetic Resource Exchange (Los Angeles, CA, USA) and the National Institutes of Mental Health (Bethesda, MD, USA) center for collaborative genomic studies on mental disorders. These biorepositories use a gold-standard examination to diagnose AD, either the autism diagnostic observation schedule (ADOS) or the autism diagnostic interview revised (ADI-R).

In our previous studies^65,80–84^, these LCLs where categorized into two different types of AD LCLs; ones with atypical mitochondrial respiration (AD-A) and those with typical respiration (AD-N). These metabolic groupings have been shown to be consistent and repeatable in our previous studies^65,80–84^. Twelve pairs of AD-N and AD-A LCLs were run with an age-matched male CNT LCL. The sample size chosen was based on our previous studies. CNT LCLs were derived from healthy white male donors with no documented behavioral or neurological disorder and without any first-degree relative suffering from a medical disorder that might involve mitochondrial dysfunction [mean (SD) age 8.5 (2.8) years]. CNT LCLs were obtained from Coriell Cell Repository (Camden, NJ, USA). Due to low availability of CNT LCLs which fit our criteria, a single CNT LCL was matched with two AD LCLs in one case and with three AD LCLs in another case (see Table 1). Also, five AD-A LCLs were matched twice with AD-N LCLs. This matching was done to control for variations in the measurement of mitochondrial function. On average, cells were studied at passage 12, with a maximum passage of 15. Genomic stability is very high at this low passage number. The cells were maintained in RPMI 1640 culture medium with 15% FBS and 1% penicillin/streptomycin (Invitrogen, Grand Island, NY, USA) in a humidified incubator at 37 °C with 5% CO_2_.

**Table 1:** Lymphoblastoid cell lines used in this study

Controls			AD-N subgroup			AD-A subgroup		
Cell ID	Source	Age (y)	Cell ID	Source	Age (y)	Cell ID	Source	Age (y)
GM09622	Coriell	7	02C10618	NIMH	7	03C14441	NIMH	7
GM17255	Coriell	6	02C10054	NIMH	6	01C08594	NIMH	7
GM15862	Coriell	11	04C26296	NIMH	10	03C16499	NIMH	11
GM09659	Coriell	4	04C24363	NIMH	4	1393306	AGRE	3
GM10153	Coriell	10	00C04757	NIMH	10	939303	AGRE	11
GM11626	Coriell	13	AU008404	AGRE	13	1165302	AGRE	13
GM11973	Coriell	7	02C09650	NIMH	7	02C09713	NIMH	7
GM18054	Coriell	5	03C15992	NIMH	5	01C08495	NIMH	4
GM16007	Coriell	12	05C38988	NIMH	12	1165302	AGRE	13
GM11599	Coriell	9	AU038804	AGRE	8	03C14441	NIMH	7
GM10153	Coriell	10	AU1267302	AGRE	10	03C16499	NIMH	11
GM10153	Coriell	10	03C17237	NIMH	10	939303	AGRE	11

Three types of cell lines were used with two types of autistic disorder cell lines which were characterized in our previous studies. All cell lines were derived from male participants

*Coriell* Coriell Cell Repository (Camden, NJ, USA), *NIMH* National Institutes of Mental Health Biorepository (Bethesda, MD, USA), *AGRE* Autism Genetic Resource Exchange Biorepository (Los Angeles, CA, USA)

### Seahorse assay

**Fig. 1:**
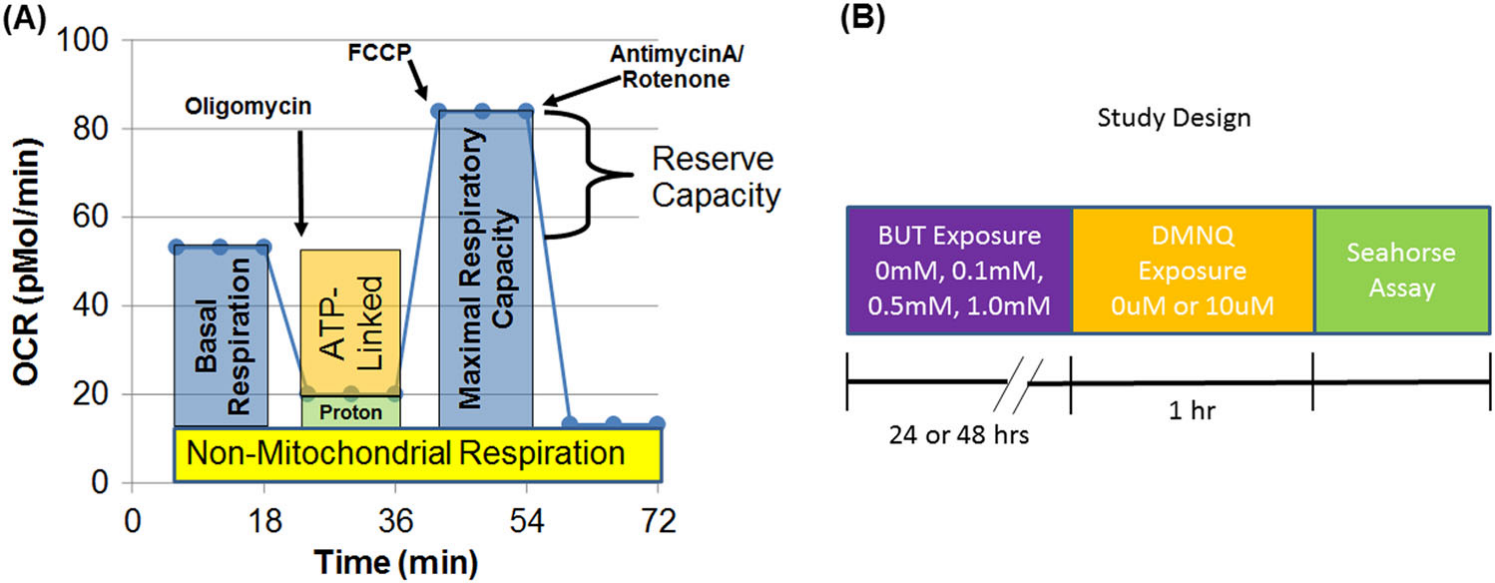
Seahorse assay and experimental timeline. (**a**) Oxygen consumption rate (OCR) is measured to determine the mitochondrial activity. Three OCRs are measured over an 18-min period to determine the mitochondrial activity. Inhibitors are added to determine several parameters of mitochondrial activity. Basal respiration is initially determined as the difference between baseline OCR and non-mitochondrial OCR. Oligomycin, which is a complex V inhibitor, is added to determine the portion of basal respiration which is ATP-linked respiration and proton leak respiration. Carbonyl cyanide-p-trifluoromethoxyphenyl-hydrazon (FCCP), a protonophore, is added to collapse the inner membrane gradient, driving the mitochondria to respire at its maximal rate. This can be used to determine maximal respiratory capacity. Antimycin A and rotenone, which are inhibitors of complexes III and I, are added to stop mitochondrial respiration in order to determine the non-mitochondrial respiration. Reserve capacity is calculated as the difference between basal respiration and maximal respiratory capacity. (**b**) Timeline for the experiment. Lymphoblastoid cell lines (LCLs) are exposed to one of the three concentrations of butyrate or not exposed as a baseline control. Following butyrate exposure, the LCLs are exposed to DMNQ for 1 h in order to increase reactive oxygen (ROS) species or not exposed to increased ROS. The Seahorse assay is performed after these exposures

A Seahorse Extracellular Flux (XF) 96 Analyzer (Seahorse Bioscience, Inc., North Billerica, MA, USA) measured the oxygen consumption rate (OCR), an indicator of mitochondrial respiration, in live intact LCLs in real-time. Each run (each line of Table 1) contained the three matched groups (CNT, AD-N, AD-A) on the same plate to control for experimental variation in the mitochondrial activity measurement. The assay (Fig. 1a), which has been described previously, provides measures of ATP-linked respiration, proton leak respiration, maximal respiratory capacity, and reserve capacity. In addition, the extracellular acidification rate (ECAR), a reflection of lactate production, is also measured during the assay. From the ECAR, glycolytic rate and glycolytic reserve are calculated. In addition, to obtain a measure of the relative utilization of oxidative vs. glycolytic pathways, two measures are calculated. The oxidative to glycolytic ratio is calculated as basal OCR divided by basal ECAR and the maximal oxidative capacity to glycolytic ratio is calculated as the maximal respiratory capacity divided by the maximal glycolytic rate.

### Redox challenge

To determine the change in mitochondrial response to BT in the context of oxidative stress, LCLs were exposed to 0 μM or 10 μM of 2,3-dimethoxy-1,4-napthoquinone (DMNQ; Sigma-Aldrich, St. Louis, MO, USA) for 1 h at 37 °C in a non-CO_2_ incubator prior to the Seahorse assay, similar to our previous studies (See Fig. 1b). A 5-mg/mL DMNQ solution was diluted in DMEM XF assay media into a 10× stock and added to cells in an XF-PS plate.

### Butyrate exposure

LCLs were cultured with one of the three BT concentrations (0.1, 0.5, and 1 mM) for 24 h or 48 h prior to the Seahorse assay or left untreated (0 mM) (Fig. 1b). These concentrations were chosen as standards in the field and simulate the concentrations in the colon but are below those known to induce apoptosis^85,86^. In humans, BT concentration is highest in the cecum, where most anaerobic gut fermentation takes place (24.5 mM/kg), and lowest in the ileum (2.3 mM/kg)^87^. BT produced from the gut microbiome is absorbed primarily from the gut into the portal vein where its concentration is about 29 µM^87^. The sodium butyrate used in this study was buffered with sodium bicarbonate in the culture medium to prevent changes in pH which could cause pH-dependent changes in the influx of BT^88^.

### Gene expression

Total RNA was isolated from 5 million LCLs using the RNeasy mini kit (Qiagen, Hilden, Germany) following manufacturer’s protocol. Complementary deoxyribonucleic acid (cDNA) synthesis (2 µg per 20 µL reaction mix) was performed using the High Capacity cDNA Reverse Transcription Kit (Applied Biosystems, Waltham MA, USA) as indicated by the manufacturer. Primers were designed using online Real time polymerase chain reaction (PCR) tool from IDT DNA (www.idtdna.com/scitools/Applications/RealTimePCR/). Table S1 outlines the primer sequences. Quantitative PCR reactions were performed for all target genes using the Power SYBR Green PCR Master Mix (Applied Biosystems, Waltham, MA) on an ABI 7900HT Fast Real Time PCR system. Relative quantification was performed for the housekeeping gene, HPRT1 (hypoxanthine phosphoribosyltransferase1).

### Analytic approach

To analyze group effects, a mixed-model regression was conducted via SAS version 9.3 (Cary, NC, USA) ‘glmmix’ procedure. The mixed model allowed data from the matched samples on each Seahorse plate to be compared to one another. The mitochondrial parameters were response variables with a between-group effect (e.g., AD-N vs. AD-A vs. CNT) and within-group repeated factors of BT concentration, exposure duration, and ROS exposure as well as the interaction between these effects. For all models, random effects included the intercept. *F*-tests were used to evaluate significance. *F*-tests from the mixed models are presented in Supplementary Tables.

Planned post-hoc orthogonal contrasts, which are *t*-distributed, were used to examine significant group and interaction effects. These results are reported as *p*-values in the graphs. When the effect of BT concentration was significant, the specific BT concentrations were compared to baseline (i.e., 0 BT) to determine if they were significantly higher or lower than the baseline. Data were normally distributed and variation was similar across the groups. Standard error bars are provided in graphs. Gene expression was analyzed similarly, although only two BT concentrations for 24 h exposure without DMNQ exposure were examined.

To examine whether the influence of BT on mitochondrial respiration was dependent on individual baseline variation in mitochondrial respiration, we used a curvilinear model with a second-order polynomial to model BT concentration as a continuous variable. Interactions with baseline mitochondrial respiration as well as interaction with exposure time and LCL group (CNT, AD-A, AD-N) were included in the model. To evaluate the influence of LCL group and the interaction of LCL group on the curve shape, the significance of the change in the log-likelihood of the model was assessed using a *χ*^2^ distribution^89^. The model effects are reported in Table S3. Planned orthogonal contrasts were conducted to determine the differences among the LCL groups when the effects were significant. Goodness-of-fit of the models was assessed using *r*^2^ and *F*-ratios. The models fit well to the data [ATP-linked respiration: *F*(8405) = 137.8, *p* < 0.0001, *r*^2^ = 0.63, *r* = 0.79; proton leak respiration: *F*(8405) = 36.3, *p* < 0.0001, *r*^2^ = 0.40, *r* = 0.63; maximum respiratory capacity: *F*(13,400) = 76.7, *p* < 0.0001, *r*^2^ = 0.60, *r* = 0.77; reserve capacity: *F*(13,400) = 52.01, *p* < 0.0001, *r*^2^ = 0.41, *r* = 0.64]. Resulting graphs display individual LCLs response to BT based on predicted curves using the model parameters and individual LCLs data. Curves are the average of the 24-h and 48-h exposures since these were not significantly different in the model.

## Results

We examined three different aspects of the effect of BT. First, we analyzed the group differences between ASD and CNT groups with respect to the effect of BT and increased ROS exposure. Second, we examined how individual baseline mitochondrial function influences the effect of BT. Lastly, we studied the ability of BT to regulate the expression of the key genes.

### Group differences of the effect of BT on ASD and CNT cell lines

We approach the comparison between CNT and ASD cell lines and the differences between the two types of ASD cell lines (AD-N, AD-A) in a systematic fashion. First, we examined the effect of BT on CNT LCLs in the absence of DMNQ exposure. Second, we examined the effect of DMNQ (i.e., increase in ROS) on CNT LCLs. We then used these findings from CNT LCLs as a baseline to compare the effect of BT on the two groups of ASD LCLs. We then examine the effect of ROS exposure (i.e., pretreatment with DMNQ) on ASD LCLs in reference to the CNT LCLs. Table S2 provides the *F*-values for the effect of the mixed model in this section. Results of individual comparisons are provided in the figures.

### Group differences: the effect of BT on CNT cell lines

**Fig. 2:**
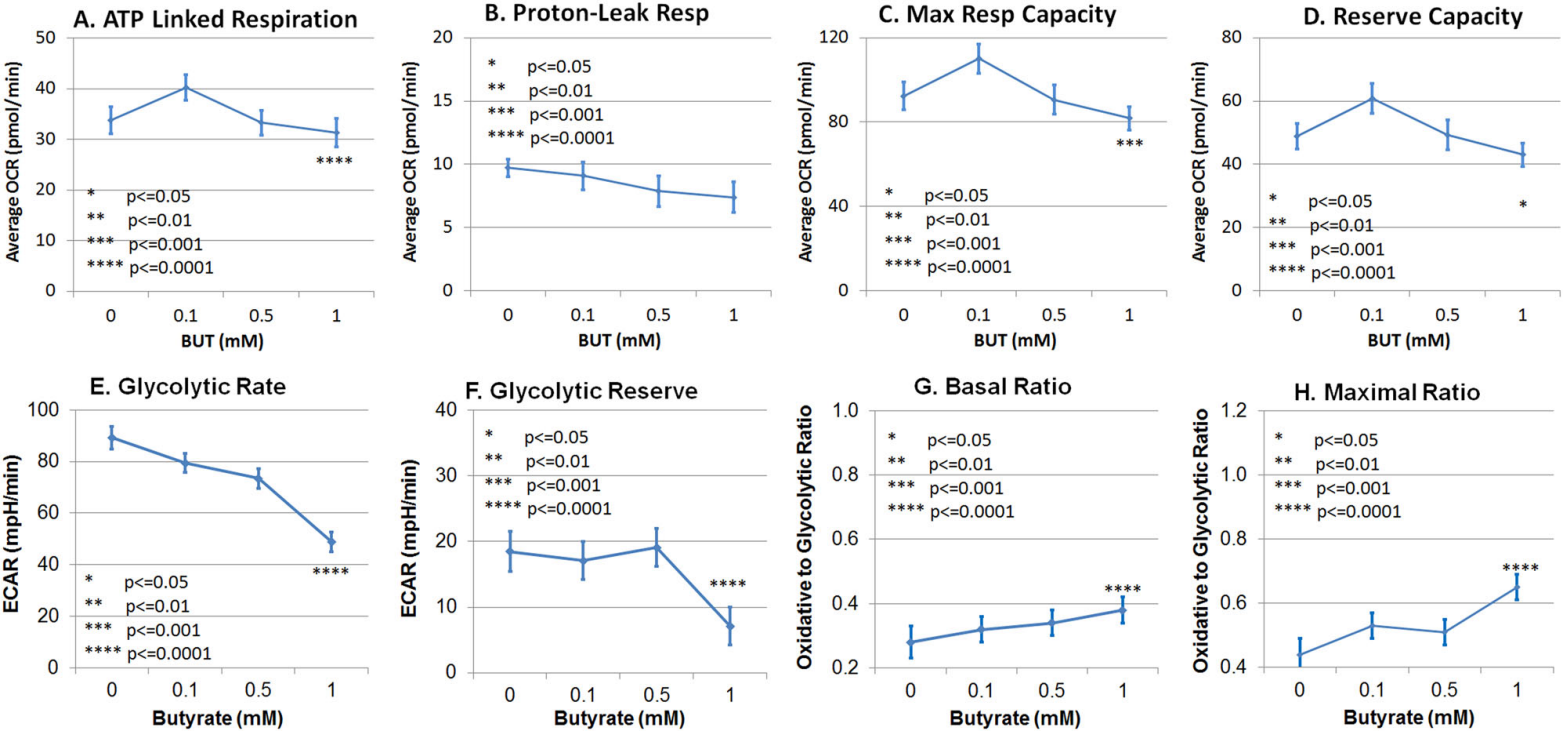
Butyrate (BT) modulates mitochondrial function in control lymphoblastoid cell lines in a concentration-dependent manner. Lymphoblastoid cell lines were exposed for either 24 h or 48 h in BT and the mitochondrial function was measured. Average changes in the mitochondrial function across the two exposure times is shown since there was no difference across the two exposure times. ATP-linked respiration (**a**), maximal respiratory capacity (**c**), reserve capacity (**d**), glycolytic rate (**e**), and glycolytic Reserve (**f**) were reduced at 1.0 mM BT relative to no BT exposure. Glycolytic metabolism was reduced to a greater extent as compared to oxidative metabolism, resulting in an increase in the basal oxidative (OCR) to glycolytic (ECAR) ratio (**g**) and the maximal oxidative (OCR) to glycolytic (ECAR) capacity ratio (**h**) for 1.0 mM BT as compared to no butyrate exposure. Since there was few difference in these relationships across BT exposure times, the average of the two exposure times is presented. Significance levels: *p ≤ 0.05, **p ≤ 0.01, ***p ≤ 0.001, ****p ≤ 0.0001

One millimolar BT reduced all the parameters of ATP production, including ATP-linked respiration, maximal respiratory capacity, and reserve capacity (Fig. 2a, c, d) but did not affect proton leak respiration (Fig. 2b). Likewise, 1 mM BT reduced glycolytic rate (Fig. 2e) and glycolytic reserve (Fig. 2e, f). The basal and maximal oxidative to glycolytic ratios (OCR/ECAR) were increased by 1 mM BT (Fig. 2g, h).

Although not shown in the figure, shorter BT exposure time (i.e., 24 h) was associated with a significantly higher oxidative to glycolytic ratio (OCR/ECAR) [mean (SE) 24 h 0.37 (0.04) vs. 48 h 0.29 (0.04)] and maximal oxidative to glycolytic capacity ratio (OCR/ECAR) [mean (SE) 24 h 0.59 (0.06) vs. 48 h 0.47 (0.06)]. No other parameters were affected by exposure time, so the figure represents an average of the two exposure times.

### Group differences: the effect of ROS on CNT cell lines

**Fig. 3:**
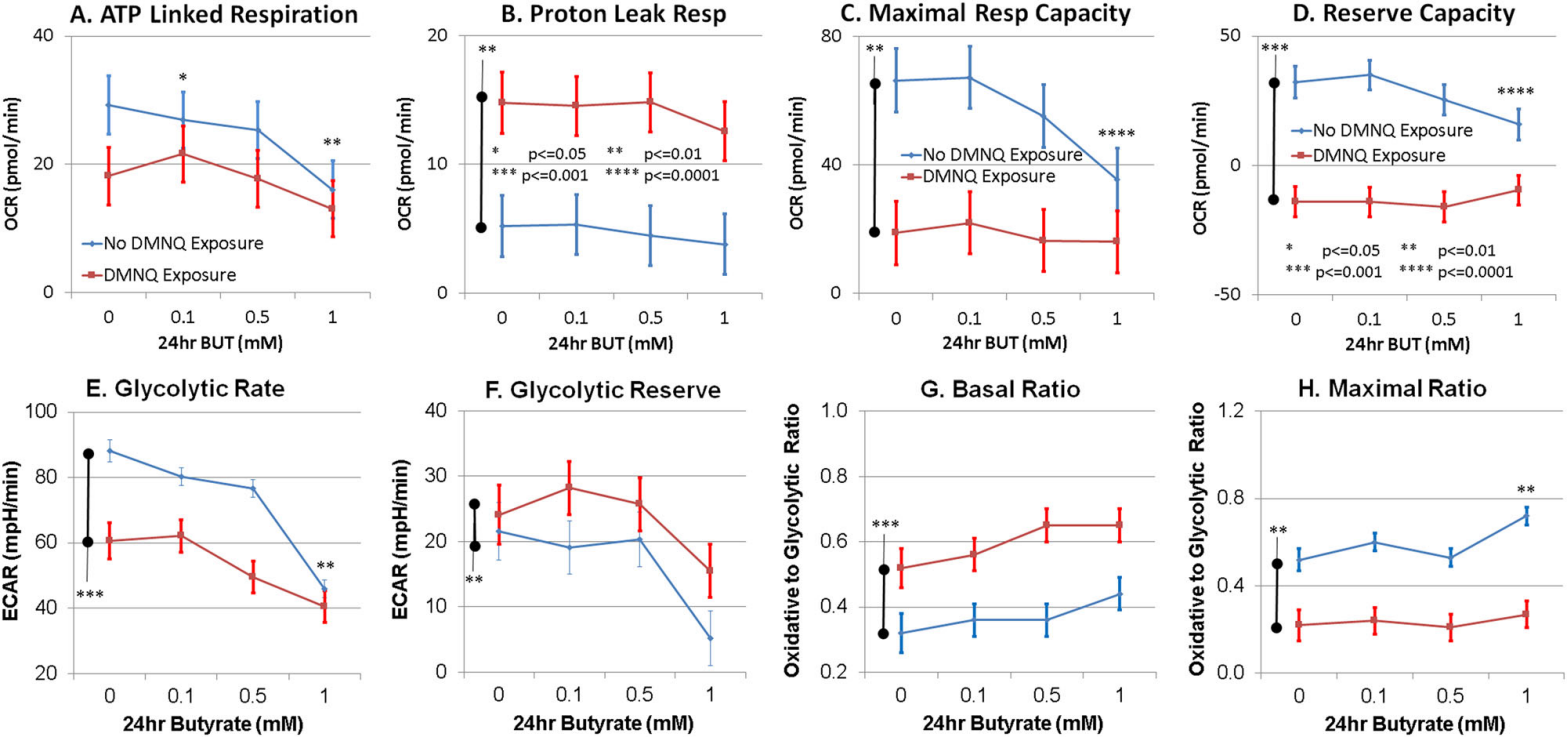
Oxidative stress overall reduced the parameters of ATP production and increases the parameters of reactive oxygen species control in mitochondrial function in control lymphoblastoid cell lines. This resulted in a decrease in reserve capacity (**d**) with butyrate (BT) exposure. Oxidative stress also reduced the glycolytic rate (**e**) but increased the glycolytic reserve capacity (**f**). Overall oxidative stress shifts the metabolism toward a more oxidative state (**g**) but decreased maximum oxidative metabolism (**h**). However, relative to baseline changes in mitochondrial function without oxidative stress, 1.0 mM BT did not have the same detrimental effect on (**a**) ATP-linked respiration, (**c**) maximal respiratory capacity, and reserve capacity (**d**), suggesting that BT may have a protective effect on mitochondria when they are under physiological stress. Lymphoblastoid cell lines are pretreated with 10 µM of 2,3-dimethoxy-1,4-naphthoquinone (DMNQ) for 1 h to increase the intracellular reactive oxygen species before the mitochondrial assay after BT has been washed from the cell culture. The bars adjacent to the data lines represent the overall significant difference. Statistical significance levels: *p ≤ 0.05, **p  ≤ 0.01, ***p  ≤ 0.001, ****p ≤ 0.0001

Since the results for the 24 h and 48 h BT exposure are so similar, only the 24 h results are presented here. The result for the 48 h BT exposure can be found in the Supplementary Materials. DMNQ lowered the maximal respiratory capacity and reserve capacity (Fig. 3c, d) and increased proton leak respiration (Fig. 3b). The strong detrimental effect of 1 mM BT on ATP-linked respiration, maximal respiratory capacity, and reserve capacity noted in the previous section (i.e., without DMNQ) was not seen when the CNT LCLs were exposed to DMNQ, resulting in a DMNQ by BT concentration interactions. In fact, there was a slight increase in reserve capacity at 1 mM BT (Fig. 3d) with DMNQ exposure as compared to no DMNQ exposure.

DMNQ lowered the glycolytic rate and increased the glycolytic reserve (Fig. 3e, f). The significant drop in glycolytic rate found at 1 mM BT concentration without DMNQ exposure was not seen when the CNT LCLs were exposed to DMNQ, resulting in a DMNQ by BT concentration interaction. DMNQ increased the oxidative to glycolytic ratio (OCR/ECAR) and decreased the maximal oxidative to glycolytic capacity ratio (OCR/ECAR) (Fig. 3g, h). The increase in the maximal oxidative to glycolytic capacity ratio found at 1 mM in CNT LCLs without exposure to DMNQ was not seen with DMNQ exposure, resulting in a DMNQ by BT concentration interaction.

### Group differences: ASD cell lines

Here, we compare the difference in mitochondrial respiration between the ASD and CNT LCLs. The graphs represent the difference from respiration of control LCLs. Since the results for the two exposure times are similar, only the results for the 24 h exposure are reported here and the results for the 48 h exposures are reported in the Supplementary Materials.

### Group differences: the effect of 24 h BT exposure on ASD cell lines

**Fig. 4:**
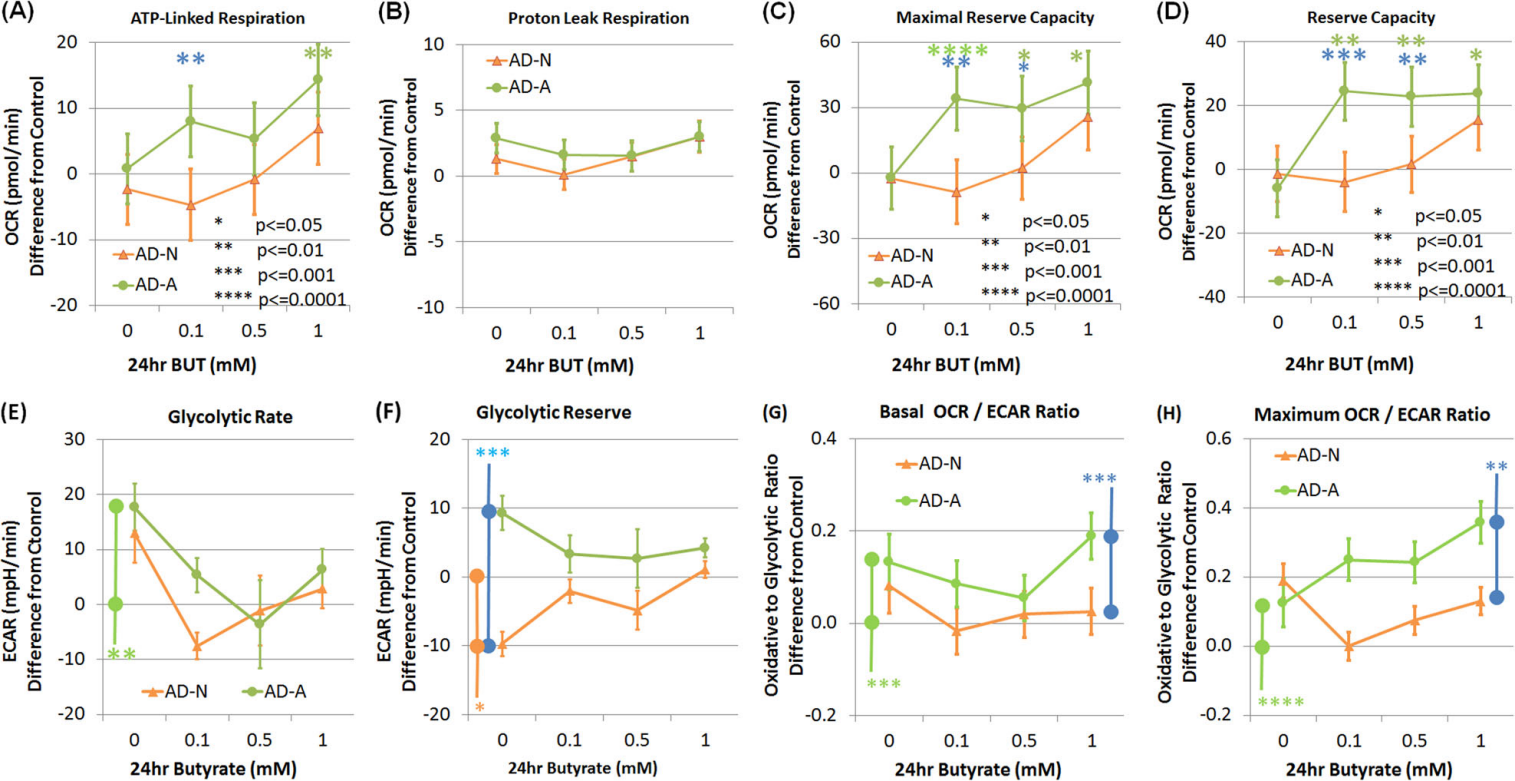
Butyrate (BT) enhances the mitochondrial function in autistic lymphoblastoid cell lines (LCLs) with atypical mitochondrial function (AD-A) over and above the effect it has on control LCLs in a concentration- and exposure time-dependent manner. ATP-linked respiration (**a**), maximal respiratory capacity (**c**), and reserve capacity (**d**) were enhanced over and above the control values for the AD-A LCLs with BT exposure, particularly for 0.1 and 1.0 mM BT concentrations. The bars adjacent to the data lines represent overall significant differences. The color of the stars and bars represents the specific comparisons. Green represents the difference between AD-A and control LCLs. Orange represents the difference between AD-N and control LCLs. Blue represents the difference between the AD-N and AD-A LCLs. Statistical significance levels: *p  ≤ 0.05, **p ≤ 0.01, ***p ≤ 0.001, ****p ≤ 0.0001

BT exposure resulted in significant increases in the respiratory parameters linked to ATP production in the AD-A LCLs as compared to the other LCL groups, resulting in a BT concentration by group interaction for ATP-linked respiration, maximal respiratory capacity, and reserve capacity. ATP-linked respiration was higher than baseline for AD-A at 0.1 mM BT as compared to AD-N and at 1 mM BT as compared to CNT (Fig. 4a). Maximal respiratory capacity was higher than baseline for AD-A at 0.1 and 0.5 mM BT as compared to CNT and AD-N and at 1 mM BT as compared to CNT (Fig. 4c). Reserve capacity increased from baseline for AD-A at 0.1 and 0.5 mM BT as compared to CNT and AD-N, and at 1 mM BT as compared to CNT (Fig. 4d).

Glycolytic rate of AD-A was significantly higher than CNT LCLs (Fig. 4e). AD-N demonstrated a significantly lower glycolytic reserve as compared to AD-A (Fig. 4f). The oxidative to glycolytic ratio (OCR/ECAR) was significantly higher for AD-A as compared to AD-N and CNT (Fig. 4g). The maximal oxidative to glycolytic capacity ratio (OCR/ECAR) was significantly higher in AD-A as compared to AD-N and CNT (Fig. 4h).

### Group differences: the effect of oxidative stress on 24 h BT exposure on ASD cell lines

**Fig. 5:**
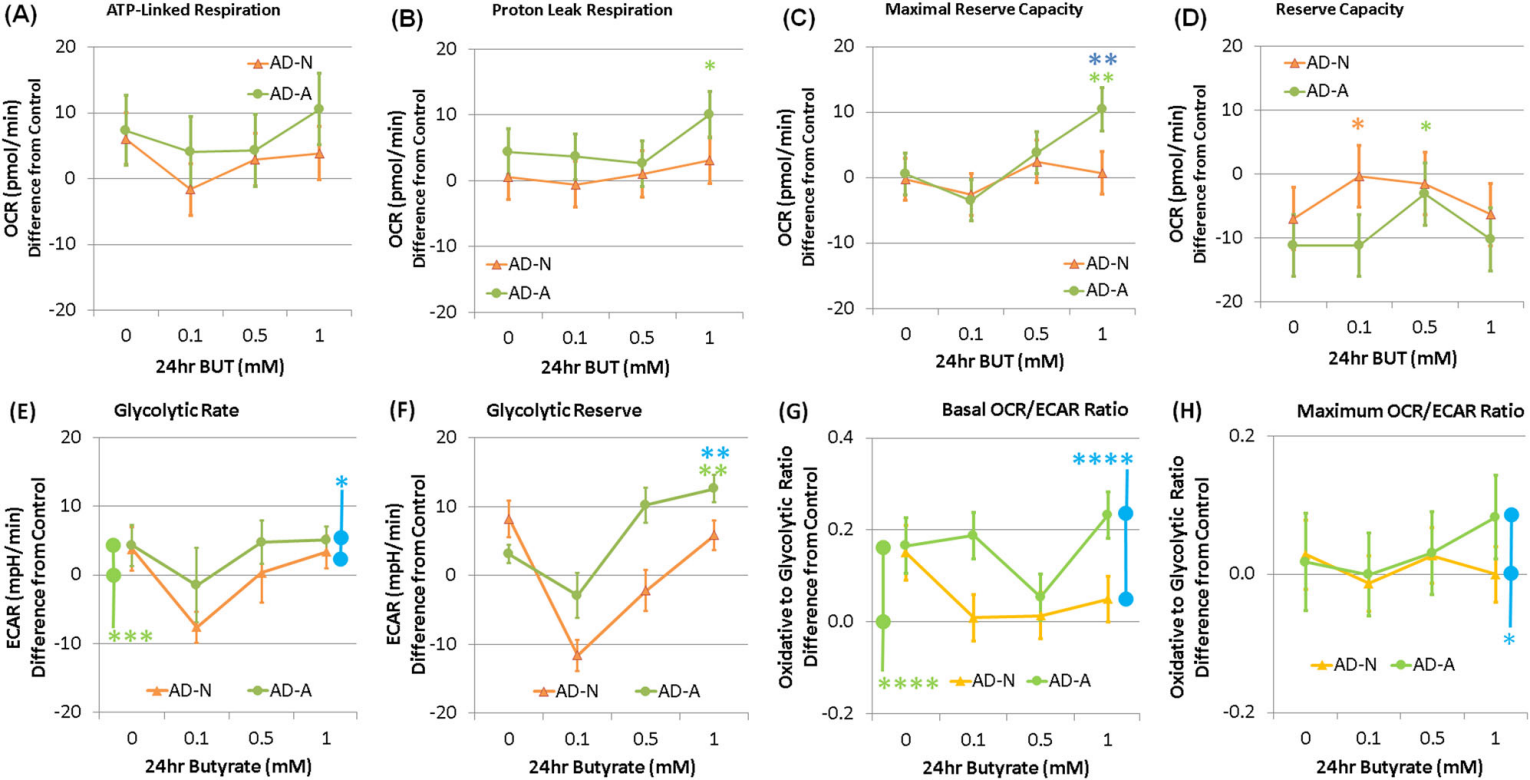
Butyrate (BT) enhances the parameters of ATP production even when the ASD lymphoblastoid cell lines (LCLs) are subjected to increased oxidative stress, especially for the ASD LCLs with atypical mitochondrial function (AD-A). LCLs are pretreated with 10 µM of 2,3-dimethoxy-1,4-naphthoquinone (DMNQ) for 1 h to increase the intracellular reactive oxygen species before the mitochondrial assay after BT has been washed from the cell culture. BT significantly increased the maximal respiratory capacity (**c**) above the control values in a manner similar to the increase seen without DMNQ exposure. This positively affected the reserve capacity (**d**). The color of the stars and bars represents the specific comparisons. Green represents the difference between AD-A and control LCLs. Orange represents the difference between AD-N and control LCLs. Blue represents the difference between the AD-N and AD-A LCLs. Statistical significance levels: *p ≤ 0.05, **p ≤ 0.01, ***p ≤ 0.001, ****p ≤ 0.0001

Maximal respiratory capacity, reserve capacity, and proton leak respiration demonstrated BT concentration by group interactions. Some results paralleled the 24 h BT exposure without DMNQ. Maximal respiratory capacity was higher for AD-A at 1 mM BT as compared to CNT and AD-N (Fig. 5c). Reserve capacity was significantly higher for AD-A at 0.5 mM BT as compared to CNT and for AD-N at 0.1 mM as compared to CNT (Fig. 5d). Proton leak respiration was significantly higher for AD-A at 1 mM BT as compared to CNT (Fig. 5b).

Glycolytic rate was significantly higher for AD-A as compared to CNT and AD-N (Fig. 5e). For glycolytic reserve, there was a group by BT concentration interaction due to AD-A being able to maintain glycolytic reserve with an exposure of 1 mM BT as compared to AD-N (*p* < 0.01) and CNT where glycolytic reserve was lost (Fig. 5f). Oxidative to glycolytic ratio (OCR/ECAR) was higher for AD-A as compared to AD-N and CNT (Fig. 5g). Maximal oxidative to glycolytic capacity ratio (OCR/ECAR) was significantly higher for AD-A as compared to AD-N (Fig. 5h).

### Individual differences: the effect of butyrate on variation in respiratory function

**Fig. 6:**
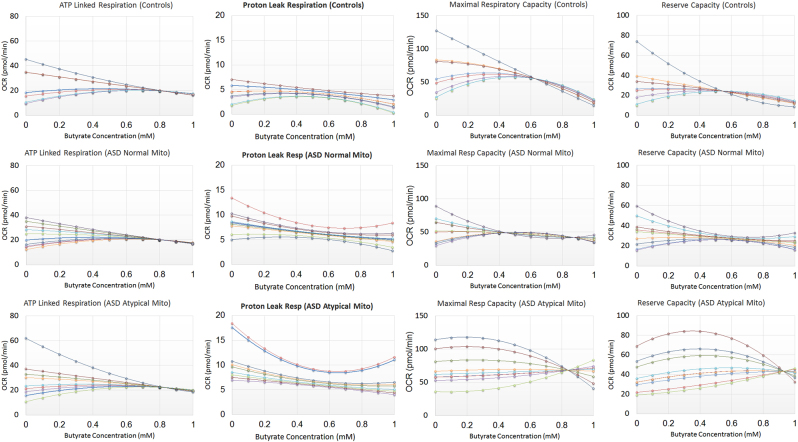
The effect of butyrate on the individual variation in mitochondrial function for the three types of lymphoblastoid cell lines

The effect of BT on ATP-linked respiration was found to be curvilinear with the intercept different across LCL groups because the AD-A curve intercept was higher than the AD-N (*p* < 0.0001) and CNT (*p* < 0.0001) intercepts. Individual baseline ATP-linked respiration influenced the effect of BT on ATP-linked respiration (Fig. 6). BT regulated ATP-linked respiration such that ATP-linked respiration was modulated to a particular set-point regardless of the baseline ATP respiration. LCLs with baseline ATP-linked respiration values higher than the set-point were decreased toward the set-point with increasing BT concentrations while LCLs with ATP-linked respiration values lower than the set-point increased toward the set-point with increasing BT concentrations. This effect appears to occur on a continuum such that higher concentrations of BT (0.7–1.0 mM) had the most profound effect.

The effect of BT on proton leak respiration was found to be curvilinear with the intercept different across LCL groups because the CNT intercept was lower than the AD-N (*p* < 0.001) and AD-A (*p* < 0.01) intercepts. Individual baseline proton leak respiration influenced the effect of BT on proton leak respiration (Fig. 6). BT reduced proton leak respiration with this effect more marked for LCLs with a higher baseline proton leak respiration. This effect occurred on a continuum such that medium concentrations of BT (0.5 mM to 0.7 mM) had the most profound effect with higher BT concentrations resulting in an increase in proton leak respiration variation.

The effect of BT on maximal respiratory capacity was found to be curvilinear with the curve of the AD-A group significantly different as compared to the CNT (*p* < 0.0005) and AD-N (*p* < 0.005) groups. Baseline maximal respiratory capacity influenced the effect of BT on maximal respiratory capacity with this effect significantly different for the AD-A group as compared to the CNT (*p* < 0.0005) and AD-N (*p* < 0.005) groups (Fig. 6). BT regulated maximal respiratory capacity for CNT and AD-N groups such that maximal respiratory capacity was modulated to a particular set-point as BT concentration increased. LCLs with baseline maximal respiratory capacity higher than the set-point decreased toward the set-point with increasing BT concentration while LCLs with maximal respiratory capacity lower than the set-point increased toward the set-point with increasing BT concentration. For the CNT and AD-N groups this effect occurred on a continuum with moderate concentrations of BT (0.4 mM to 0.6 mM) having the optimal effect and higher concentrations (>0.6 mM) resulting an overall decrease in maximal respiratory capacity. For the AD-A group, the effect of BT is slightly different than the other groups. Although BT regulates maximal respiratory capacity to a set-point, this occurs at higher BT concentrations (0.8 mM) with the set-point being overall higher. This explains the mean data in Fig. 4 as the maximal respiratory capacity for AD-A LCLs is maintained at a higher value while the maximal respiratory capacity for the CNT LCLs was lower at higher BT concentrations.

The effect of BT on reserve capacity was found to be curvilinear with the curve of the AD-A group significantly different as compared to the CNT (*p* < 0.001) and AD-N (*p* = 0.001) groups. Baseline reserve capacity influenced the effect of BT on reserve capacity with this effect significantly different in the AD-A group as compared to the CNT (*p* < 0.001) and AD-N (*p* = 0.001) groups (Fig. 6). For CNT and AD-N LCLs, reserve capacity was modulated by BT concentration to a particular set-point despite the reserve capacity baseline. LCLs with baseline reserve capacity higher than the set-point decreased toward the set-point with increasing BT concentration while LCLs with reserve capacity lower than the set-point increased toward the set-point as BT concentration increased. This effect occurred on a continuum with moderate concentrations of BT (~0.6 mM) having the optimal effect and higher BT concentrations (>0.6 mM) resulting in a lower in reserve capacity for CNT LCLs and a more variable reserve capacity for the AD-N LCLs. For the AD-A LCLs, BT regulated reserve capacity to an overall higher set-point at higher BT concentration (~0.9 mM). This explains the mean data in Fig. 4 where the reserve capacity for AD-A LCLs is maintained at a higher value while the reserve capacity for the CNT LCLs was lower at higher BT concentrations.

### Gene expression studies: BT alters the expression of mitochondrial genes

**Fig. 7:**
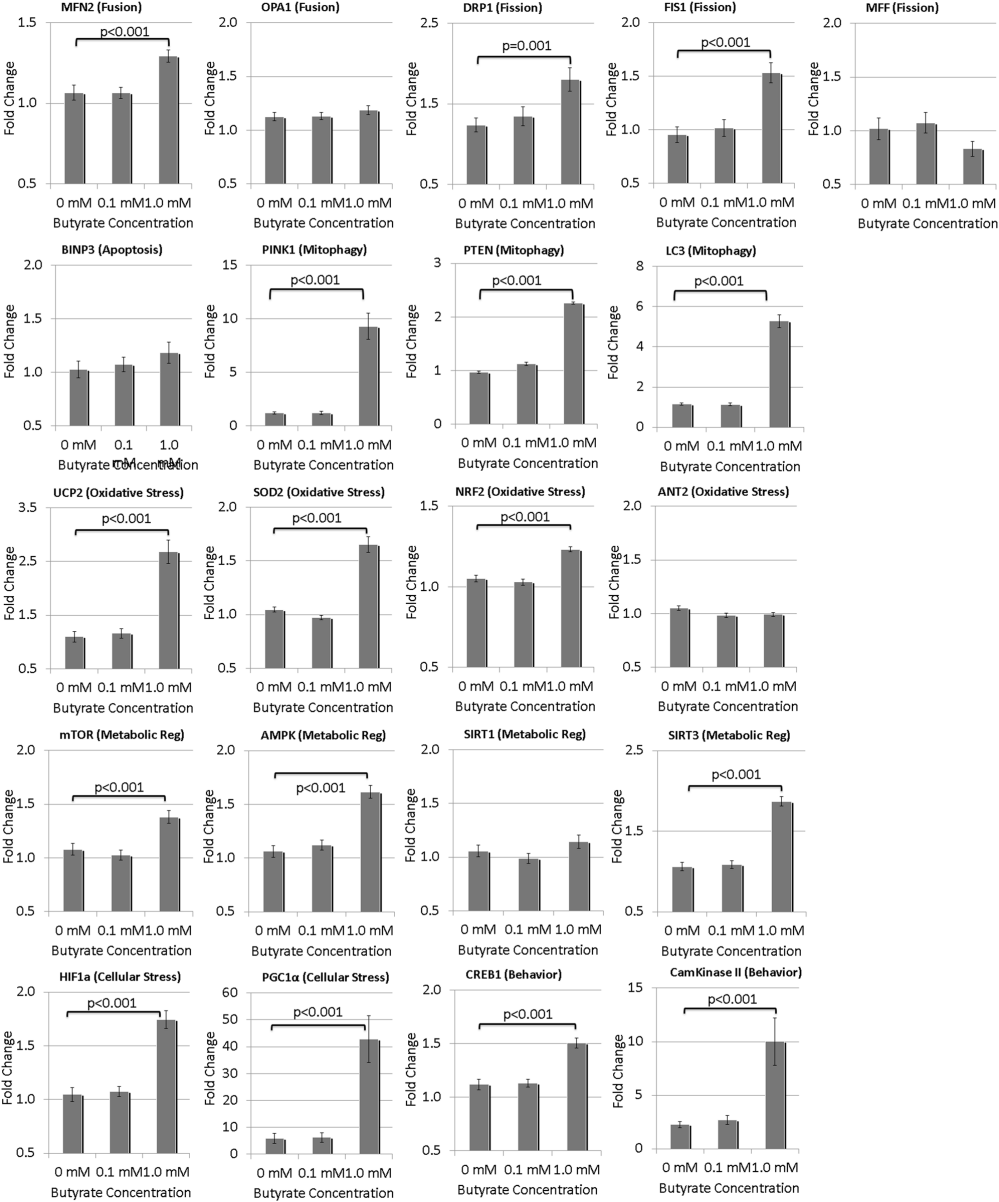
Change in expression of key genes involved in the response to physiological stress as a result of butyrate exposure

We determined whether 0.1 mM and 1.0 mM BT altered gene expression as compared to baseline (0 mM BT) across the three LCL groups (CNT, AD-N, AD-A). Overall we found no LCL groups by BT concentration interaction, but did find differences in gene expression as a result of BT concentration and across LCL groups separately (see Table S4 for statistical values). BT significantly increased the expression of many genes at 1 mM, but not at 0.1 mM (Fig. 7).

BT resulted in an increase in the expression of genes associated with mitochondrial dynamics, particularly mitochondrial fission and mitophagy (Fig. 7). Two of the three mitochondrial fission genes (DRP1, FIS1) showed a significant increase. All of the three mitophagy genes showed a significant increase, specifically PINK1, LC3, and PTEN. Only one of the two fusion genes, specifically MFN2, demonstrated a modest increase. The one apoptosis gene studied (BNIP3) did not significantly increase in expression. Three of the four genes associated with response to oxidative stress increased expression, specifically UCP2, NRF2, and SOD2. Three of the four genes associated with energy metabolism increased expression, specifically mTOR, AMPK, and SIRT3. The two genes associated with physiological stress demonstrated significant upregulation, specifically PGC1α and HIF1α. In addition, the pathways associated with the regulation of behavior and cognition were also upregulated, including Cam Kinase II and CREB1.

Several differences in gene expression were found between LCL groups independent of the BT concentration. AD-N demonstrated significantly greater expression than CNT in several genes including Cam Kinase II (*p* = 0.01), DRP1 (*p* < 0.01), MFN2 (*p* < 0.01), mTOR (*p* < 0.05), and OPA1 (*p* < 0.01). AD-A LCLs demonstrated significantly greater expression than CNT in several genes including CREB (*p* < 0.01) and DRP1 (*p* = 0.01). Additionally, as compared to AD-A, AD-N LCLs showed lower expression of HIF1α (*p* = 0.01) and higher expression of SIRT1 (*p* < 0.05).

## Discussion

This is the first systematic investigation of the effects of BT on mitochondrial function in LCLs as well as the first study to demonstrate the effect of BT on mitochondria in tissues from individuals with ASD. In this study, we examined the effect of BT on mitochondrial function in transformed B-cells (i.e., LCLs) derived from children with ASD with and without a unique type of mitochondrial dysfunction as well as LCLs from typically developing age-matched boys. Further to both the positive and negative effects of SCFAs including BT in various conditions^90^, it was hypothesized that several factors such as BT concentration, exposure time, microenvironment redox state, and cell type would alter the manner in which BT is metabolized and utilized. Thus, these parameters were systematically altered to gain an insight into the effects of BT on mitochondrial function.

Although the measurements of SCFA concentrations are technically difficult^27^, it should be noted that the BT concentrations selected were physiologically relevant. The highest concentration of BT, equivalent to 1000 µM BT, is consistent with inter-enteric concentration in the colon but much higher than the systemic concentrations. The lowest concentration of BT, which is equivalent to 100 µM, is closer to blood concentrations in the portal vein^87^. Portal vein concentrations are about twice the hepatic vein concentrations due to the metabolism of BT in the liver, and peripheral blood concentrations are about one-third the hepatic vein concentrations^87^. Thus, under normal circumstances, the highest level of BT we examined are only relevant to enteric physiology in healthy individuals, although disruptions of the microbiome could increase these levels beyond the typical concentration during dysbiotic states. However, BT may act locally to alter GI physiology and/or the gut-associated lymphoid tissue (GALT) function, which may, in turn, affect host physiology. Below we summarize our findings as well as discuss the potential importance of BT in health and disease.

### The effect of BT on control cell lines

BT demonstrated a concentration-dependent effect on CNT LCLs such that the highest BT concentration influenced mitochondrial function with this effect independent of the exposure time. One millimolar BT decreased the respiratory parameters linked to ATP production, including ATP-linked respiration, maximal respiratory capacity, and reserve capacity, but did not have any effect on the regulation of ROS at the inner mitochondrial membrane. BT decreased glycolysis in the control LCLs, with a greater decrease in glycolytic metabolism as compared to oxidative metabolism, resulting in an increase in the oxidative to glycolytic ratios.

Increasing ROS reversed this detrimental effect of BT on respiratory parameters in CNT LCLs, actually increasing ATP-linked respiration at 0.1 mM BT and increasing reserve capacity at 1.0 mM BT in the presence of ROS. Interestingly, BT had a more complex effect on glycolytic pathways, reducing glycolytic rate but increasing glycolytic reserve. This resulted in a complex change in the relative oxidative to glycolytic pathways, resulting in a relative increase in the basal ongoing oxidative respiration but a decrease in the relative maximal oxidative respiration.

The analysis of individual LCLs provides some insight into the effect of oxidative stress on the mitochondria in the CNT LCLs. Indeed, in the individual LCL analysis, BT was found to modulate the mitochondrial function toward a set-point unique to the LCL group such that high values are brought down toward the set-point and low values are brought up to the set-point. Thus, this suggests that BT may help optimize the mitochondrial function specific to the physiology of the particular cell. This highlights the positive effect of BT on cells under physiological stress. The potential mechanisms are discussed below.

### The effect of BT on LCLs derived from children with ASD

For AD-A LCLs, BT increased the respiratory parameters related to ATP production, including ATP-linked respiration, maximal respiratory capacity, and reserve capacity, as compared to baseline. This effect was not seen for the AD-N LCLs. This effect of increasing the respiratory parameters cannot be simply interpreted as positive or negative, but the DMNQ challenge which increased the intracellular oxidative stress can help put these findings into context.

AD-A LCLs at baseline are found to have increases in respiratory parameters related to ATP production. Although we believe that this is an adaptation to protect the cell and might make AD-A LCLs more resistant to toxicants^84^, in our previous studies, we found that the AD-A LCLs are more sensitive to acute increases in ROS. Indeed, in other studies, without BT exposure, challenge with DMNQ to acutely increase ROS results in a significant decrease in respiratory parameters related to ATP production in AD-A LCLs as compared to control and AD-N LCLs^80,81,83^. However, in this study, when AD-A LCLs were exposed to BT for 24 or 48 h, treatment with DMNQ to increase ROS increased the respiratory parameters linked to ATP production to an equal amount or, in many cases, a higher amount than the control LCLs. This is consistent with our recent study where we demonstrated that treatment with DMNQ after prolonged exposure to a toxicant enhanced the respiratory parameters related to ATP production in AD-A but not AD-N LCLs^84^. This effect was believed to occur due to the recruitment of mitoplasticity pathways, resulting in the upregulation of genes to improve mitochondrial function during times of physiological stress such as the ones identified to be upregulated in this study.

AD-N LCLs, which do not show mitochondrial dysfunction at baseline, were not significantly influenced by BT except when ROS was increased, in which case reserve capacity was significantly elevated at 0.1 mM BT. This again suggests that BT has a modulatory effect on the mitochondria by recruiting pathways involved in enhancing mitochondrial function to protect the cell from physiological stressors.

### Effects of BT on mitochondrial protective pathways

BT has been shown to have positive health effects by stimulating mitochondrial function. In high fat diet-induced obese mice, BT enhanced the mitochondrial oxidative phosphorylation and upregulated the expression of fatty acid oxidation enzymes and uncoupling proteins (UCP)^69^ and induced nuclear-encoded mitochondrial genes, including peroxisome proliferator-activated receptor-gamma coactivator-1 alpha (PGC1α), resulting in mitochondrial adaptations that result in more complete β-oxidation and improved insulin sensitivity^70,71^. Many of these changes occur as part of a process known as mitoplasticity, which refers to the mitochondria’s ability to adapt in order to optimally function in the face of changes in energy demand, substrate availability, pathophysiology, or environmental stressors^91^. Mitoplasticity involves pathways that modulate general cellular energetics, mitochondrial enzymes in the citric acid cycle (CAC) and electron transport chain (ETC), proton leak, redox regulation, and transcription factors as well as genes that regulate the control of mitochondrial repair and regeneration such as mitochondrial biogenesis, mitophagy, and mitochondrial fission and fusion^91^. Many of these mitoplastic processes may occur through PGC1α as PGC1α upregulates genes important for mitochondrial metabolism, including nuclear respiratory factors (NRF-1, NRF-2), UCP, and genes essential for redox metabolism, including superoxide dismutase, catalase, and glutathione peroxidase-1 and decreases expression of genes that inhibit ETC function (AKT)^92,93^. PGC1α also has the effect of upregulating ETC complex genes as well as mitochondrial DNA transcription. Such effects may account for the increases in ATP-linked respiration and maximal respiratory capacity.

Our data suggest that BT upregulates the genes involved in mitoplasticity, particularly genes involved in response to physiological stress (PGC1α, HIF1α), mitochondrial fission (DRP1, FIS1), mitophagy (PINK1, LC3, PTEN), oxidative stress (UCP2, NRF2, SOD2) as well as energy metabolism (mTOR, AMPK, SIRT3). Our study demonstrates that the increases in expression of these genes occurs at 1.0 mM BT but not at lower BT concentrations. At this concentration, BT increased the oxygen consumption and attenuated glycolysis, particular in the AD-A LCLs. Interestingly, this same pattern of modulation of mitochondrial function has been shown in other cell types which exhibit mitochondrial dysfunction, specifically breast cancer cells where BT has an anti-cancer effect^51^.

Proton leak respiration represents a mechanism to compensate for increased ROS at the inner mitochondrial membrane, which is mediated by UCP2 in lymphocytes. UCP2 is known to be upregulated in the context of prolonged oxidative stress and serves to protect the mitochondria^94,95^. We have shown that UCP2 is increased in the AD-A LCLs as compared to AD-N LCLs^80^. Interestingly, BT has been shown to reduce mitochondrial ROS by increasing proton leak through upregulation of UCP2^96^. We have shown that upregulation of UCP2 is an important mechanism for regulating ROS in AD-A LCLs in previous studies^80,81^, in ASD siblings with biomarkers of increased ROS^81^, and have now demonstrated that BT further increases the gene expression of UCP2 in all LCLs regardless of the type in this study, so BT may be enhancing this compensatory mechanism in the AD-A LCLs.

In cell lines other than LCLs, BT also seems to positively modulate the mitochondrial integrity. For example, BT improved the survival of Chinese hamster ovary cells by recruitment of a mitophagy protein, Parkin, along with inducing autophagic removal of damaged mitochondria^97^. In our study, we found that the genes associated with mitophagy and recruitment of genes involved in pathways which typically compensate for physiological stress, such as DRP1, are upregulated with increased BT concentrations.

### Effects of BT on genes involved in the regulation of learning, memory, and behavior

In this study, we show that BT increases the expression of genes involved in behavior and learning, specifically CREB and CamKinase II. Such findings are consistent with our previous studies, which showed enhanced CREB and phospho-CREB immunoreactivity in brains from rats centrally infused with PPA^60^, and similar findings with PPA and BT exposure in CREB-associated pathways in rat pheochromocytoma cell lines^33^. Many findings relating to CREB physiology in the brain have been shown to have parallels in peripheral immune cells^98,99^. This is a subject of further study. We have proposed that enteric SCFAs may provide a functional pathway for the microbiome to “enforce memory” in the host nervous system, and that over exposure of these metabolites may lead to the enhanced memory, rigid behavior, and motor symptoms associated with ASD by increasing CREB expression^60^. CamKinase II activity has been proposed to be a mechanism associated with regulating repetitive, involuntary, and obsessive behaviors in psychiatric disease^100^, suggesting that BT could have a potential role in modulating these ASD-associated behaviors.

### Connection with patients with autism

There are many lines of evidence that BT may be helpful in normalizing the behavior and physiological abnormalities in ASD. In animal models of ASD, BT positively modulates neurotransmitter gene expression^67^, and rescues the ASD-type behavior^66^ and brain pathology^66,68^ induced by prenatal valproic acid exposure^66^. Additionally, BT has been found to modulate the ASD-related genes in cell line models^33^. BT has interesting effects on behavior and gene expression in the brain of the BTBR mouse model of ASD. BT decreased the excitatory and increased the inhibitory neurotransmitter genes in the prefrontal cortex and had a positive effect on behavior by increasing social behavior and decreasing repetitive behavior through modulating the excitatory–inhibitory balance of the brain^67^. Interestingly, BT is helpful in other neurological disorders that have an underlying mitochondrial dysfunction as it has been shown to alleviate cognitive deficits in premotor stage Parkinson’s disease^41^. Thus, BT may be a promising treatment for children with ASD. Further preclinical and clinical studies will be needed to investigate this possibility.

### Connection with the enteric microbiome

Studies point to overrepresentation of *Clostridia* spp in children with ASD^101–105^, particularly children with ASD who experienced developmental regression^59,106^ and/or those with GI symptoms at or before ASD symptom onset^58^. Treatment with vancomycin, an antibiotic aimed at decreasing *Clostridia* spp, transiently decreases ASD symptoms^107^ and studies have suggested that the reduction in dietary refined carbohydrates^108^, a substrate for enteric bacteria that produce PPA, result in ASD symptom improvement. In addition, our previous studies have suggested that an overabundance of PPA, a SCFA produced by *Clostridia* spp, could be disrupting mitochondrial function in some children with ASD^37^. This suggests that *Clostridia* spp, or other SCFA producing gut bacteria, could have a connection with the etiology of ASD.

However, the same bacteria which produce PPA also produce BT, the relative production of which may be dependent upon the dietary substrate. We have also shown that high intracerebroventricular infusions of BT produce hyperactivity and some lipid effects consistent with ASD, similar to that found with PPA infusions, albeit to a lesser extent^109,110^. Furthermore, BT has been shown to upregulate genes for fatty acid oxidation. Interestingly, the increased acyl-carnitines seen in the PPA rat model of ASD and as well as in children with ASD suggest a reduced ability to breakdown fatty acids^37^. Thus, it may be that there is an imbalance in the relative production of BT in relation to PPA or in the bacteria that preferentially produce BT, particularly at key neurodevelopmental time periods associated wiht the development of ASD. Furthermore, various low refined carbohydrate diets proposed to help in ASD may do so by reducing these substrates leading to reduced SCFA production by ASD-associated bacteria, while supplementation of foods higher in complex fibers (i.e., inulins) may exert a therapeutic response by preferentially increasing BT production over PPA^18^.

BT is absorbed both passively and actively and is transported by specific monocarboxylate transporters which also transport other SCFAs (i.e., propionate) and ketones^111^. BT also activates specific free fatty acid G protein-coupled receptors^7^. However, BT is also a mitochondrial fuel that is directly integrated into energy metabolism through the production of Acetyl-CoA, the first metabolite of the CAC. In fact, germ-free mice demonstrate a specific deficit in bioenergetics in colon tissue with this deficiency rescued by the BT-producing bacteria *Butyrivibrio fibrisolvens*. Further experiments have suggested that this effect was a consequence of BT being used as a mitochondrial fuel rather than its effect as a histone deacetylase inhibitor^40^.

### Limitations

It should be noted that the findings in this study may or may not relate to other tissue types (i.e., brain, colon), doses, or critical time periods in development. Additionally, BT has other indirect effects, including the activation of broad signaling pathways such as those that are induced by free fatty acid G protein-coupled receptors activation, which can exert complex effects on immunity, mitochondrial function, and gene expression independent of the direct effects of BT on mitochondrial function and gene expression noted here. We do not know if these effects are independent, reinforcing, mutually exclusive, or compensatory. These are a subject of further inquiry.

Human studies, particularly clinical trials, will be most helpful in further understanding if BT is therapeutic for human disease. Indeed, although we have generally interpreted the results to demonstrate a positive effect of BT on physiology, biological organisms are complex, so the theoretical interpretation of the data may not be a direct application to complex intact physiological systems. Additionally, some of the alterations in gene function may not be beneficial in all populations. For example, individuals with Tuberous Sclerosis, a disorder characterized by uncontrolled upregulation of mTOR, would worsen with further upregulation of mTOR.

Nonetheless, these findings are consistent with the effects of BT and other SCFA and microbiome metabolites, such as PPA, as having potential to broadly modulate host physiology and behavior in both health and disease, in particular ASD.

## Conclusion

BT is an SCFA derived from the enteric microbiome which is a mitochondrial fuel and modulator of mitochondrial function and gene expression. In this study, we demonstrated, for the first time, that BT has a modulatory effect on mitochondrial function in LCLs from both healthy children and children with ASD. What is most significant about our study is that we found that BT has a positive effect on a cell line under physiological stress, including cell lines with baseline normal mitochondrial function that are induced to have mitochondrial dysfunction through inducing physiological stress and, in particular, cell lines with underlying mitochondrial dysfunction (i.e., AD-A). This ability to rescue cells under physiological stress may provide insight into the therapeutic effect of BT in many disease states and may suggest that BT may be a novel therapeutic agent that may have implications for many diseases associated with mitochondrial dysfunction as well as patients with mitochondrial disease.

Further research will be needed to extend these findings to other cell types (i.e., neurons, glia, liver, gut) and other disease states. Nonetheless, this study provides a new understanding of how the enteric microbiome may modulate host physiology at a variety of levels and may contribute to the etiology and possible treatment of ASD and other diseases.

## Electronic supplementary material

Supplementary Results

Table S1

Table S2

Table S3

Table S4

Figure S1
