# Supplementary material for: Butyrate enhances mitochondrial function during oxidative stress in cell lines from boys with autism
Source: Transl Psychiatry. 2018 Feb 2;8:42. doi: 10.1038/s41398-017-0089-z (PMC5804031; doi:10.1038/s41398-017-0089-z)
Supplement: Supplementary file 1 — Supplementary Results [file 41398_2017_89_MOESM1_ESM.docx]

Group Differences: The Effect of Reactive Oxygen Species on CNT Cell Lines Exposed to Butyrate for 48 hrs.

DMNQ lowered ATP-Linked Respiration, Maximal Respiratory Capacity and Reserve Capacity (Figure S1A,C,D) and increased Proton Leak Respiration (Figure S1B). DMNQ by Concentration interaction was found for Reserve Capacity. This interactions were driven because 1mM BT did not have the same detrimental effect on mitochondrial respiratory parameters in CNTl LCLs after being exposed to DMNQ as compared to not being exposed to DMNQ. In fact there was an increase in Reserve Capacity at 1mM BT (Figure S1D) compared to no DMNQ treatment.

DMNQ lowered glycolytic rate (Figure S1E) and increased glycolytic reserve (Figure S1F). DMNQ by Concentration interaction was found for glycolytic rate (Figure S1E). This interaction was due to the glycolytic rate dropping to a greater extent at 1mM BT concentration without DMNQ exposure as compared to with DMNQ exposure.

DMNQ increased the oxidative to glycolytic ratio (Figure S1G) and decreased the maximal oxidative to glycolytic capacity ratio (Figure S1H).

Group Differences: The Effect of 48 hour BT Incubation on Autism Cell Lines

AD-A LCLs demonstrated a significant increase in respiratory parameters linked to ATP production with 48hr BT exposure, similar to the findings for the 24hr BT exposures. Concentration by Group interactions were found for ATP-Linked Respiration, Maximal Respiratory Capacity and Reserve Capacity.

ATP-Linked Respiration was significantly higher than baseline for AD-A at 1mM BT as compared to CNT and AD-N. Maximal Respiratory Capacity was significantly higher than baseline for AD-A at 0.1 mM BT as compared to CNT and at 1mM BT as compared to CNT and AD-N. Reserve Capacity was significantly higher than baseline for AD-A at 0.1 mM BT as compared to CNT and AD-N, at 0.5mM BT as compared to CNT and AD-N and at 1mM BT as compared to CNT and AD-N. BT did not affect Proton Leak Respiration differently across the LCL groups.

Glycolytic rate was significantly different across LCL groups because CNT [Mean (SE) 71.6 (2.7)] had a lower glycolytic rate as compared to AD-N [t(290)=2.69, p<0.01; Mean (SE) 76.9 (3.6)] and AD-A [t(290)=1.96, p=0.05; Mean (SE) 75.3 (3.7)]. Glycolytic reserve was significantly different across groups since AD-A LCLs [Mean (SE) 20.4 (1.9)] demonstrated an higher glycolytic reserve as compared to AD-N [t(290)=3.99,p<0.0001; Mean (SE) 15.1 (1.8)] and control [t(290)=3.27, p=0.001; Mean (SE) 15.9 (1.9)].

Oxidative to glycolytic ratio was significantly different across LCL groups because AD-A [Mean (SE) 0.40 (0.04)] demonstrated a higher ratio as compared to AD-N [t(290)=4.82,p<0.0001; Mean (SE) 0.33 (0.04)] and CNT [t(290)=6.38, p<0.0001; Mean (SE) 0.30 (0.04)]. The maximal oxidative to glycolytic capacity was significantly different across LCL groups because AD-A LCLs [Mean (SE) 0.63 (0.06)] demonstrated a significantly higher ratio as compared to AD-N [t(290)=3.49, p<0.001; Mean (SE) 0.54 (0.06)] and CNT [t(290)=5.66, p<0.0001; Mean (SE) 0.48 (0.06)] and AD-N LCLs demonstrated a significantly higher ratio as compared to CNT [t(290)=2.31, p<0.05].

Group Differences: The Effect of Elevated Oxidative Stress and 48 hour BT Incubation on Autism Cell Lines

Concentration by Group interactions were found for ATP-Linked Respiration and Maximal Respiratory Capacity. Some results paralleled the 48hr BT exposure without DMNQ. As compared to baseline, ATP-Linked Respiration was increased for AD-A at 0.5mM BT as compared to CNT and at 1mM BT as compared to CNT and AD-N. As compared to baseline, Maximal Respiratory Capacity was increased for AD-A at 1mM BT as compared to AD-N and at 0.5 mM BT as compared to CNT.

Glycolytic rate was significantly different across LCL groups because CNT [Mean (SE) 49.3 (3.5)] demonstrated a lower glycolytic rate as compared to AD-A [t(303)=4.84, p<0.0001; Mean (SE) 56.0 (3.4)] and AD-N [t(303)=7.04, p<0.0001; Mean (SE) 59.0 (3.4)]. There was a significant group by BT concentration interaction. This interaction occurred because AD-A were better able to maintain glycolytic rate near baseline as BT concentration increased as compared to the other groups which demonstrated reductions in glycolytic rate as BT concentration increased. This resulted in a significant different between AD-A and AD-N at 0.1mM [t(303)=2.76, p<0.01], 0.5mM [t(303)=3.60, p<0.0005] and 1.0mM [t(303)=2.43,p<0.05] BT and CNT at 0.1 BT [t(303)=2.62, p<0.01]. Glycolytic reserve capacity was not different across LCL groups.

Oxidative to glycolytic ratio was significantly different across LCL groups because AD-A [Mean (SE) 0.67 (0.07)] demonstrated a significantly higher ratio as compared to AD-N [t(303)=4.25, p<0.0001; Mean (SE) 0.59 (0.07)] and CNT [t(303)=4.38, p<0.0001; Mean (SE) 0.59 (0.07)]. The maximal oxidative to glycolytic capacity ratio was not significantly different across LCL groups.
