# Supplementary material for: Butyrate enhances mitochondrial function during oxidative stress in cell lines from boys with autism
Source: Transl Psychiatry. 2018 Feb 2;8:42. doi: 10.1038/s41398-017-0089-z (PMC5804031; doi:10.1038/s41398-017-0089-z)
Supplement: Supplementary file 2 — Table S1 [file 41398_2017_89_MOESM2_ESM.docx]

| Table S1. List of primer sequences used for real time polymerase chain reaction analysis. F: Forward. R: Reverse. | | |
| --- | --- | --- |
| Genes | NLM ID | Primer sequences |
| HPRT1 | NM_000194 | F: 5' - TGCTGAGGATTTGGAAAGGG - 3' |
|  |  | R: 5' - ACAGAGGGCTACAATGTGATG - 3' |
| UCP2 | NM_003355 | F: 5' - TCCTGAAAGCCAACCTCATG - 3' |
|  |  | R: 5' - GGCAGAGTTCATGTATCTCGTC - 3' |
| MTOR | NM_004958 | F: 5' - CAAGAACTCGCTGATCCAAATG - 3' |
|  |  | R: 5' - GCTGTACGTTCCTTCTCCTTC - 3' |
| PINK1 | NM_032409 | F: 5' - GAGTATGGAGCAGTCACTTACAG - 3' |
|  |  | R: 5' - CAGCACATCAGGGTAGTCG - 3' |
| MFN2 | NM_014874 | F: 5' ATGTGGCCCAACTCTAAGTG 3' |
|  |  | R:5' CACAAACACATCAGCATCCAG 3' |
| CREB1 | NM_004379 | F: 5' TTCTACAGTATGCACAGACCAC 3' |
|  |  | R: 5' ATGCCATAACAACTCCAGGG 3' |
| HIF1A | NM_001530 | F: 5' AACATAAAGTCTGCAACATGGAAG 3' |
|  |  | R: 5' TTTGATGGGTGAGGAATGGG 3' |
| OPA1 | NM_015560 | F: 5' GGAGAACCATATTCGTTTTGACC 3' |
|  |  | R: 5' AGAGCTGTTCCCTTTTCCTG 3' |
| CAMK2B | NM_001220 | F: 5' AGACTTCGGCCTAGCTATCG 3' |
|  |  | R: 5' GTACAGGATCACCCCACATG 3' |
| PGC1 (PPARGC1A) | NM_013261 | F: 5' CAGGCAGTAGATCCTCTTCAAG 3' |
|  |  | R: 5' TCCTCGTAGCTGTCATACCTG 3' |
| BNIP3 | NM_004052 | F: 5' GGGATCTATATTGGAAGGCGTC 3' |
|  |  | R: 5' CCAGGATCTAACAGCTCTTCAG 3' |
| DNM1L (DRP1) | NM_005690 | F: 5' TTCCATTATCCTCGCTGTCAC 3' |
|  |  | R: 5' CATCAGTACCCGCATCCATG 3' |
| FIS1 | NM_016068 | F: 5' TGACATCCGTAAAGGCATCG 3' |
|  |  | R: 5' CTTCTCGTATTCCTTGAGCCG 3' |
| MFF | NM_020194 | F: 5' TAAATGAGTAAAGGAACAAGCAGTG 3' |
|  |  | R: 5' AGCAGTGGGAGAAGGAAATG 3' |
