# Supplementary material for: Butyrate enhances mitochondrial function during oxidative stress in cell lines from boys with autism
Source: Transl Psychiatry. 2018 Feb 2;8:42. doi: 10.1038/s41398-017-0089-z (PMC5804031; doi:10.1038/s41398-017-0089-z)
Supplement: Supplementary file 3 — Table S2 [file 41398_2017_89_MOESM3_ESM.docx]

| Table S2. Mixed-model statistic results for Seahorse Assay. Only significant values are provided | | | |
| --- | --- | --- | --- |
| **Control Cell Lines without an increase in oxidative stress** | | | |
| Seahorse Parameter | Concentration | Exposure Time | Interaction |
| ATP-Linked Respiration | F(3,133)=11.65, p<0.0001 |  |  |
| Maximal Respiratory Capacity | F(3,133)=7.56, p=0.0001 |  |  |
| Reserve Capacity | F(3,133)=4.42, p=0.005 |  |  |
| Proton Leak |  |  |  |
| Glycolytic rate | F(3,160)=54.24,p<0.0001 |  |  |
| Glycolytic reserve | F(3,160)=19.48,p<0.0001 |  |  |
| Oxidative to glycolytic ratio | F(3,160)=7.76, p<0.0001 | F(1,160)=24.30, p<0.001 |  |
| Maximal oxidative to glycolytic capacity | F(3,160)=14.87, p<0.0001 | F(1,160)=28.31, p<0.001 |  |
| **Control Cell Lines with an increase in oxidative stress for 24hr Exposu**re | | | |
|  | Concentration | DMNQ | Interaction |
| ATP-Linked Respiration |  |  | F(3,124)=3.19,p=0.02 |
| Maximal Respiratory Capacity |  | F(1,8)=13.80, p=0.006 | F(3,124)=10.58, p<0.0001 |
| Reserve Capacity |  | F(1,8)=26.44, p<0.0009 | F(3,124)=13.03, p<0.0001 |
| Proton Leak |  | F(1,8)=14.75, p<0.005 |  |
| Glycolytic rate |  | F(1,8)=31.82, p=0.0005 | F(3,128)=6.71, p<0.0005 |
| Glycolytic reserve |  | F(1,8)=11.06, p=0.01 |  |
| Oxidative to glycolytic ratio |  | F(1,8)= 25.15,p=0.001 |  |
| Maximal oxidative to glycolytic capacity |  | ); F(1,8)= 34.66, p<0.01 | [F(3,128)=4.03,p<0.01 |
| **Control Cell Lines with an increase in oxidative stress for 48hr Exposure** | | | |
|  | Concentration | DMNQ | Interaction |
| ATP-Linked Respiration |  | F(1,10)=4.77,p=0.05 |  |
| Maximal Respiratory Capacity |  | F(1,10)=33.15, p=0.0002 |  |
| Reserve Capacity |  | F(1,10)=95.90, p<0.0001 | F(3,154)=3.43,p=0.02 |
| Proton Leak |  | F(1,10)=56.38, p<0.0001 |  |
| Glycolytic rate |  | F(1,10)=69.98, p<0.0001 | F(3,168)=3.58,p=0.01 |
| Glycolytic reserve |  | F(1,10)=5.07, p=0.05 |  |
| Oxidative to glycolytic ratio |  | F(1,10)= 88.10,p<0.0001 |  |
| Maximal oxidative to glycolytic capacity |  | F(1,10)= 40.01,p<0.0001 |  |
| **Autism Cell Lines without an increase in oxidative stress for 24hr Exposure** | | | |
|  | Concentration | Group | Interaction |
| ATP-Linked Respiration |  |  | F(6,199)=2.10, p=0.05 |
| Maximal Respiratory Capacity |  |  | F(6,199)=2.67, p<0.05 |
| Reserve Capacity |  |  | F(6,199)=2.80, p=0.01 |
| Proton Leak |  |  |  |
| Glycolytic rate |  | F(2,208)=3.89,p<0.05 |  |
| Glycolytic reserve |  | F(2,208)=7.77,p<0.001 |  |
| Oxidative to glycolytic ratio |  | F(2,208)=9.81, p<0.0001 |  |
| Maximal oxidative to glycolytic capacity |  | F(2,208)=10.96, p<0.0001 |  |
| **Autism Cell Lines without an increase in oxidative stress for 48hr Exposure** | | | |
|  | Concentration | Group | Interaction |
| ATP-Linked Respiration |  |  | F(6,198)=2.35, p<0.05 |
| Maximal Respiratory Capacity |  |  | F(6,198)=2.27, p<0.05 |
| Reserve Capacity |  |  | F(6,198)=2.33, p<0.05 |
| Proton Leak |  |  |  |
| Glycolytic rate |  | F(2,290)=3.76,p<0.05 |  |
| Glycolytic reserve |  | [F(2,290)=9.03, p<0.0005 |  |
| Oxidative to glycolytic ratio |  | F(2,290)=22.08, p<0.0001 |  |
| Maximal oxidative to glycolytic capacity |  | F(2,290)=16.29, p<0.0001 |  |
| **Autism Cell Lines with an increase in oxidative stress for 24hr Exposure** | | | |
|  | Concentration | Group | Interaction |
| ATP-Linked Respiration |  |  |  |
| Maximal Respiratory Capacity |  |  | F(6,256)=3.89, p=0.001 |
| Reserve Capacity |  |  | F(6,256)=2.43, p<0.05 |
| Proton Leak |  |  | F(6,256)=3.02, p<0.01 |
| Glycolytic rate |  | F(2,254)=6.46,p<0.005 |  |
| Glycolytic reserve |  |  | F(6,254)=2.14,p<0.05 |
| Oxidative to glycolytic ratio |  | F(2,254)=12.88, p<0.0001 |  |
| Maximal oxidative to glycolytic capacity |  | F(2,254)=3.14, p<0.05 |  |
| **Autism Cell Lines with an increase in oxidative stress for 48hr Exposure** | | | |
|  | Concentration | Group | Interaction |
| ATP-Linked Respiration |  |  | F(6,202)=2.35, p<0.05 |
| Maximal Respiratory Capacity |  |  | F(6,202)=2.48, p<0.05 |
| Reserve Capacity |  |  |  |
| Proton Leak |  |  |  |
| Glycolytic rate |  | F(2,303)=25.44,p<0.0001 | F(6,303)=2.86, p<0.01 |
| Glycolytic reserve |  |  |  |
| Oxidative to glycolytic ratio |  | F(2,303)=12.76, p<0.0001 |  |
| Maximal oxidative to glycolytic capacity |  |  |  |
