# Supplementary material for: Butyrate enhances mitochondrial function during oxidative stress in cell lines from boys with autism
Source: Transl Psychiatry. 2018 Feb 2;8:42. doi: 10.1038/s41398-017-0089-z (PMC5804031; doi:10.1038/s41398-017-0089-z)
Supplement: Supplementary file 4 — Table S3 [file 41398_2017_89_MOESM4_ESM.docx]

| Effect | ATP-Linked Respiration | Proton Leak  Respiration | Maximal Respiratory Capacity | Reserve Capacity |
| --- | --- | --- | --- | --- |
| Group | F(2,394)=20.66, p<0.0001 | F(2,394)=5.96, p<0.01 |  |  |
| Curve | Χ^2^(2)=9, p=0.01 | Χ^2^(2)=7.5, p<0.05 | Χ^2^(2)=11.0, p<0.005 | Χ^2^(2)=9.5, p<0.01 |
| Curve x Group |  |  | Χ^2^(2)=27.5, p<0.0001 | Χ^2^(2)=10.5, p<0.01 |
| Individuals Baseline by Butyrate Concentration | Χ^2^(4)=83, p<0.0001 | Χ^2^(4)=23, p=0.0001 | Χ^2^(4)=61.5, p<0.0001 | Χ^2^(4)=47.3, p<0.0001 |
| Individuals Baseline by Butyrate Concentration by Group |  |  | [Χ^2^(3)=11.5, p<0.01 | Χ^2^(3)=16.0, p=0.001 |
