# Supplementary material for: Butyrate enhances mitochondrial function during oxidative stress in cell lines from boys with autism
Source: Transl Psychiatry. 2018 Feb 2;8:42. doi: 10.1038/s41398-017-0089-z (PMC5804031; doi:10.1038/s41398-017-0089-z)
Supplement: Supplementary file 5 — Table S4 [file 41398_2017_89_MOESM5_ESM.docx]

| Table S3. Statistical results of Gene Expression Comparisons | | | |
| --- | --- | --- | --- |
| Gene | Group | Exposure | Interaction |
| MFN2 | F(2,36)=4.25, p<0.05 | F(2,36)=11.34, p<0.0002 |  |
| OPA1 | F(2,36)=4.20, p<0.05 |  |  |
| DRP1 | F(2,36)=4.96, p=0.01 | F(2,36)=6.89, p<0.005 |  |
| FIS1 |  | F(2,36)=15.65, p<0.0001 |  |
| MMF |  |  |  |
| BINP3 |  |  |  |
| PINK1 |  | F(2,36)=45.50, p<0.0001 |  |
| PTEN |  | F(2,36)=180.73, p<0.0001 |  |
| LC3 |  | F(2,36)=31.62, p<0.0001 |  |
| UCP2 |  | F(2,36)=40.40, p<0.0001 |  |
| SOD2 |  | F(2,36)=14.86, p<0.0001 |  |
| NRF2 |  | F(2,36)=7.36, p<0.0001 |  |
| ANT2 |  |  |  |
| mTOR | F(2,36)=3.38, p<0.05 | F(2,36)=13.63, p<0.0001 |  |
| AMPK |  | F(2,36)=32.12; p<0.0001 |  |
| SIRT1 | F(2,36)=4.89, p=0.01 |  |  |
| SIRT3 |  | F(2,36)=28.19; p<0.0001 |  |
| HIF1a | F(2,36)=3.41, p<0.05 | F(2,36)=39.26, p<0.0001 |  |
| PGC1α |  | F(2,33)=15.28, p<0.0001 |  |
| CREB1 | F(2,36)=3.95, p<0.05 | F(2,36)=26.85, p<0.0001 |  |
| CamKinase II | F(2,36)=3.46, p<0.05 | F(2,36)= 11.65, p<0.0001 |  |
