# Supplementary figures and images for: Butyrate enhances mitochondrial function during oxidative stress in cell lines from boys with autism
Source: Transl Psychiatry. 2018 Feb 2;8:42. doi: 10.1038/s41398-017-0089-z (PMC5804031; doi:10.1038/s41398-017-0089-z)

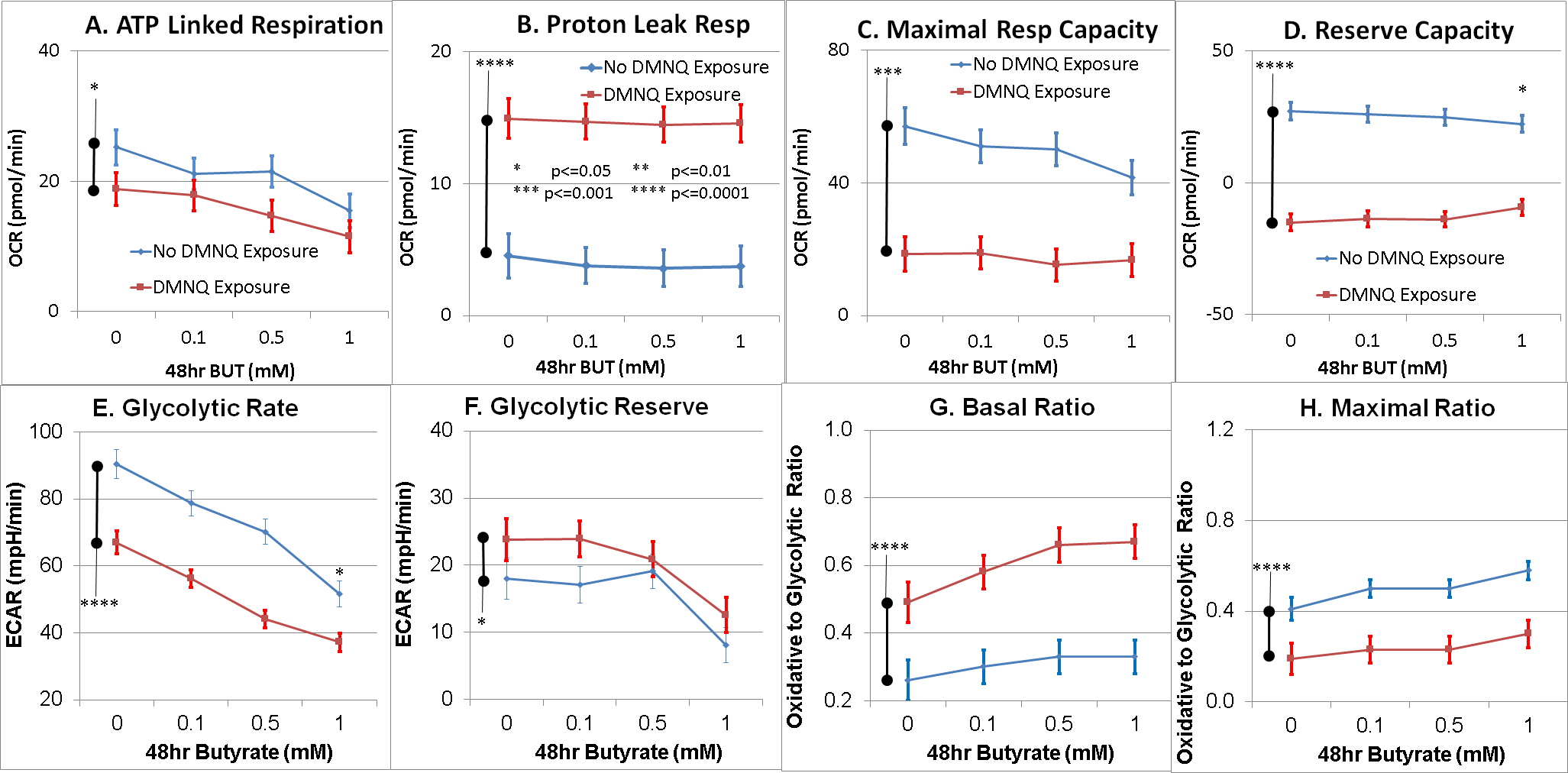

Supplement: Supplementary file 6 — Figure S1 [file 41398_2017_89_MOESM6_ESM.tif]
